# Participants' experiences of care during a randomized controlled trial comparing a lay-facilitated angina management programme with usual care: a qualitative study using focus groups

**DOI:** 10.1111/j.1365-2648.2012.06069.x

**Published:** 2012-06-28

**Authors:** Pauline Nelson, Helen Cox, Gill Furze, Robert JP Lewin, Veronica Morton, Heather Norris, Nicky Patel, Peter Elton, Richard Carty

**Affiliations:** University of ManchesterUK; York Trials Unit, University of YorkUK; Adult Nursing and Health Care, Coventry UniversityUK; University of YorkUK; NHS BuryUK; Pennine Acute NHS TrustUK

**Keywords:** cardiac rehabilitation, focus groups, lay-led care, nursing, self-management, stable angina

## Abstract

**Aim:**

This paper is a report of a qualitative study conducted as part of a randomized controlled trial comparing a lay-facilitated angina management programme with usual care. Its aim was to explore participants' beliefs, experiences, and attitudes to the care they had received during the trial, particularly those who had received the angina management intervention.

**Background:**

Angina affects over 50 million people worldwide. Over half of these people have symptoms that restrict their daily life and would benefit from knowing how to manage their condition.

**Design:**

A nested qualitative study within a randomized controlled trial of lay-facilitated angina management.

**Method:**

We conducted four participant focus groups during 2008; three were with people randomized to the intervention and one with those randomized to control. We recruited a total of 14 participants to the focus groups, 10 intervention, and 4 control.

**Findings:**

Although recruitment to the focus groups was relatively low by comparison to conventional standards, each generated lively discussions and a rich data set. Data analysis demonstrated both similarities and differences between control and intervention groups. Similarities included low levels of prior knowledge about angina, whereas differences included a perception among intervention participants that lifestyle changes were more easily facilitated with the help and support of a lay-worker.

**Conclusion:**

Lay facilitation with the Angina Plan is perceived by the participants to be beneficial in supporting self-management. However, clinical expertise is still required to meet the more complex information and care needs of people with stable angina.

## Introduction

Long-term conditions are an increasing burden on society and health services, accounting for 60% of deaths worldwide (World Health Organisation 2007). Stable angina is a long-term condition which affects approximately 2 million people in the UK and more than 50 million people world-wide, and its prevalence is growing as more people survive acute coronary events (World Health Organisation 2008, Scarborough *et al*. 2010). Over half of these people have symptoms that restrict their daily life and would benefit from knowing how to manage their condition (Fox *et al*. 2006). Research has suggested that people with long-term conditions should be involved in their own care, with self-management programmes offered to help them to gain the necessary skills (Newman 2004). However, effective self-management of a long-term condition does not simply target coping behaviour but also must address the cognitions and emotions that arise when living with a long-term condition. A systematic review of interventions to change maladaptive cognitions in people with heart disease concluded that, although the evidence base was not strong, cognitive-behavioural programmes appeared to be most successful in changing these cognitions (Goulding *et al*. 2010). To support people to self-manage, nurses need to acquire skills in cognitive-behavioural techniques which they may currently lack (Newman 2004, MacDonald *et al*. 2008).

### Background

The Angina Plan is a nurse-facilitated, home-based, cognitive-behavioural self-management programme which targets misconceptions and other maladaptive cognitions and supports behaviour change with goal setting and pacing. It includes a work-book with a diary for recording progress and a relaxation programme on CD. It is introduced to the person with angina in a 45–60 minute first interview when the principles of the programme are explained, and misconceptions about living with heart disease are dispelled. Follow-up is with four 10–15 minute consultations over 3 months, by telephone or visit. The Angina Plan was compared with routine nurse education in a randomized trial and found to improve angina report, physical and psychological functioning, and quality of life (Lewin *et al*. 2002). Although there are over 900 facilitators (mainly nurses) now trained to deliver the programme in the UK, and over 20,000 people with angina have received the programme, uptake in primary care (where the Angina Plan was originally intended to be delivered) has been disappointing.

Countries including the UK, Australia, North America, and parts of Europe have moved towards using lay-workers to deliver self-help interventions to people with long-term conditions. The underpinning rationale is the expectation that lay-led self-management will result in cost-effective health gains (Griffiths *et al*. 2007).

We set out to test if the Angina Plan could be facilitated by lay-workers overseen by the Community Cardiac Rehabilitation nursing team based on a primary care trust (PCT). The lay-workers recruited were members of the public who had, either personally or by association, some experience of cardiac disease. The lay-workers were trained over a 4-week period in facilitating the Angina Plan. All participants received advice from an angina nurse specialist following diagnosis, and people in the intervention group were visited at home by one of the three lay-workers employed by the PCT. A randomized controlled trial of the lay angina management programme (LAMP) is reported elsewhere (Furze *et al*. 2012). This article presents the nested qualitative component of the LAMP trial.

To further enhance the evidence base in health services research, current perspectives value the integrated use of a range of methods, using qualitative approaches to explore participants' beliefs and experiences (Campbell *et al*. 2000, Miller & Crabtree 2005, Craig *et al*. 2008). Focus groups bring participants together to discuss a topic in-depth, enabling detailed opinions, views and ideas to be elicited about a range of issues (Kitzinger 2006). Accordingly focus groups were planned to investigate study participants' views of both intervention and control arms of the trial and their perceptions about the usefulness/acceptability of the care experienced.

## The study

### Aim

The aim of the study was to explore, via focus groups, participants' beliefs, experiences, and attitudes to the care they had received for their angina in the randomized controlled trial, with an emphasis on those who received the lay-facilitated Angina Plan programme.

### Design

The theoretical orientation of the study was informed by the work of Krueger which offers guidelines for the design, conduct, analysis, and reporting of focus group research for applied settings (Krueger 1998a, 1998b, Krueger & Casey 2000). Over an 8-month period in 2008, four focus groups were convened with participants who had experienced care for managing their angina in the LAMP trial. Groups took place in one region of the North West of England in a meeting room provided by the local PCT. One focus group was conducted with participants receiving usual care (control), and as the focus of the study was on the angina management intervention, three focus groups were conducted with participants receiving the LAMP (intervention). Although six to eight participants have been identified as optimal for focus groups (Krueger & Casey 2000), recruitment was fairly low and groups were eventually conducted with between two and five participants. The timing of the focus groups was staggered to include participants from both earlier and later in the trial, to acknowledge that the lay-workers would gain expertise over time, which may affect participant experience. We also wished to ensure that focus groups were held reasonably near to the experience of receiving the intervention. For these reasons, it was not possible to simply wait until there were more participants for each group.

### Sampling and participants

Participants were sampled purposively on the basis of their allocated treatment group, age, and gender. Potential participants were initially approached by telephone by the trial manager based at the University of York. An information sheet detailing the procedure and venue for the focus groups was sent to those people expressing interest in participating. Those who wished to take part in the focus groups posted back the tear-off reply slip from the information sheet. In total, we invited 31 people to participate, 21 in the intervention group, and 10 in the control group. Uptake was as follows: 4/10 (40%) usual care and 10/21 (48%) intervention. Reasons for non-participation included the following: own illness, family illness, no desire to participate, unable to be contacted, family bereavement, on holiday, and unavailable on the day.

### Data collection

Focus groups were conducted in a room provided by Bury PCT, and transport costs and lunch were provided for participants. The focus groups were facilitated by a researcher with a research nurse on hand to help with practical issues. The researcher was experienced in running qualitative focus groups, with a background in health services research and a broad interest in long-term conditions self-management. However, she had no clinical training, little prior knowledge of angina, and was independent of both the clinical and research teams and thus open to and questioning of themes arising from the data.

Questions and associated prompts presented in Table 1 were developed both from the literature and a previously conducted pilot focus group and were structured into a topic guide according to a ‘questioning route’ which incorporated key questions in a planned sequence (Krueger 1998b). Participants were asked for their perceptions about the care that had been provided to them as part of the study, and their views about its usefulness and acceptability. The main topics explored were participants' beliefs about angina, their lifestyle changes, perceptions on information received, understanding of services, impressions of taking part in the study, and perceptions of lay-worker involvement. This range of topics endeavoured to capture the cognitive-behavioural focus of the intervention and the perceptions of the participants about receiving such an intervention. A focus group was held with participants from the control arm to explore perceptions of their care to assess any differences and similarities in experience.

**Table 1:** Interview topic guides

Question type		

Intervention group	Question wording	Prompts
Opening	Tell us how you found out you had angina	
Introductory	How did you come to be in the study?	
Transition	*Perceptions about the study*	
	What has it been like to be part of the study?	useful/not useful elements challenges enjoyable elements strategies/consequences
Key question 1	*Perceptions about the Angina Plan*	useful/not useful elements ease/difficulty of use enjoyable elements strategies/consequences relevant/irrelevant parts best parts improvements
	What did you think of the Angina Plan?	
Key question 2	*Perceived changes*	what/how/why?
	Have you changed anything in your life because of being in this study?	
	Are there changes you are going to make in the future because of being in the study or not?	
Key question 3	*Perceptions of the lay facilitator*	
	What did you think about getting help about your health from a non-medical person?	useful/not useful elements challenges enjoyable elements strategies/consequences relevant/irrelevant parts best parts improvements
Key question 4	*Perceptions about potential wider use*	what/how/why improvements
	Do you think this study would be useful to others with angina?	
Key question 5	*Perceptions about information*	what/how/where/whom?
	What new information have you learned from being in the study that you didn't know before?	
	Did you get information from elsewhere about your health? If so where?	
	What didn't you learn that you would've liked?	
Ending questions	What has been the most useful part of the study for you?	
	Is there anything we have missed that you would like to add?		
	
Control group	Opening, introductory, transition questions, key questions 2, 4, 5, and ending questions as above	

Key question 1	*Experiences of care*	useful/not useful elements ease/difficulty enjoyable elements strategies/consequences relevant/irrelevant parts best parts improvements
	What did you think about the care you were offered?	
	What sort of choices were you offered?	
Key question 3	*Perceptions of the cardiac rehabilitation nurse*	what/how/why?
	What did you think about getting help about your health from the nurse?	

Data were recorded with an audio recorder (Edirol R-09) and transcribed verbatim by an independent transcriber at the University of York. Field notes were written after each focus group to record impressions of group dynamics, thoughts on the functioning of questions, and initial impressions of salient issues arising from the discussion. Interview transcripts were checked and anonymized by the independent researcher who had moderated the focus groups and participants were allocated pseudonyms. As the purpose of analysis was to arrive at an interpretation of the data based on abstract, overarching themes which might be different from those of the people taking part, member checking (sharing findings with participants for their comment) was not undertaken. As noted by Goldblatt *et al*. (2011), member checking is a controversial procedure and is ‘not necessarily the best strategy for achieving credibility’ (p. 394).

### Ethical considerations

Research Ethics Committee approval was obtained by the relevant Local NHS Research Ethics Committee. Written consent was collected from all participants prior to any data collection. Participants consented to the groups being recorded and were informed that all identifiable data would be removed once transcribed. Participants were informed that they were free to withdraw from the study at any time without their care being affected and that answering questions was entirely voluntary.

### Data analysis

Anonymous transcripts were uploaded to the NVivo 7 qualitative data software package (QSR 1999). Data segments from the transcripts were coded, compared, and contrasted with other codes and grouped into more overarching themes to build up an analysis of key concepts until main data themes were saturated and no new ideas were apparent (Krueger 1998a). Field notes were used for analytic purposes to supplement the coding of transcripts. Transcripts, field notes, coding, and themes were jointly reviewed by members of the study team to question the emerging analysis and suggest alternative explanations. Findings, which were reached by consensus, are presented in the next section, using quotations from a range of participants denoted by gender and an individual code number.

### Rigour

Study rigour was enhanced through the paired use of guidelines to guide the study's design and conduct. Table 2 details how such guidelines enabled the production of detailed and credible findings about participants' experiences of angina self-management.

**Table 2:** Study rigour

Tools	Steps to enhance rigour in the study design and conduct
(a) Krueger's guidance for the design and conduct of focus group research (Krueger 1998a, 1998b, Krueger & Casey 2000)	setting relevant questions from knowledge of the prior literature eliciting views from a varied sample of participants using a series of coding steps moving from ‘open’ to more focused and abstract coding to analyse data writing of field notes to supplement analysis organizing data in a specialized computer package constant comparison and contrast of data for similarities and differences joint review of analysis by several researchers from different professional perspectives taking a reflexive stance about the researcher's influence of the data
(b) Critical Appraisal Skills Programme (CASP) guidelines for the appraisal of qualitative research (Critical Appraisal Skills Programme, 2007)	

## Results

Characteristics of the focus groups participants are presented in Table 3.

**Table 3:** Participant characteristics

Participant characteristics	Control (*n* = 4)	Intervention (*n* = 10)	Overall (*n* = 14)
Mean age (range) years	67·75	63·60	64·79
	(47–82)	(52–69)	(47–82)
Male	3	6*	9
Married	3 (+1 widowed)	10	13 (+1 widowed)
Mean (sd) episodes angina in 1 week	1·75 (1·26)	2·90 (3·64)	2·57 (3·13)
Comorbidities	1 (Type 2 DM)	0	0
Admissions for revascularization during study follow-up	1	2	3
Canadian Angina Classification			
Class 1	2	8	10
Class 2	1	1	2
Class 3	1	1	2

Canadian Angina Classification: Classification of functional limitations due to angina. People at with Class 1 experience angina on strenuous physical exertion only, whereas people with Class 4 experience angina with any exertion and may experience angina at rest.

sd, standard deviation; DM, diabetes mellitus.

* Plus one female carer.

### General impressions of focus group discussions

Group discussions lasted a mean duration of 82·31 minutes, and despite the small numbers, generated lively discussions and rich data. Participants were keen to share experiences and learn from each other, particularly about angina symptoms and how they had discovered and subsequently managed angina. Members of both the control and intervention groups taking part in the focus groups had positive views of the angina care delivered to them via the study and commonly valued the health service provision that had been made available.

#### Main themes

Participants shared stories about when they first experienced angina symptoms and their uncertainty about its identity:

I thought angina was a sharp pain, the pain going down your left arm and I didn't have any of that at all. So, it was a shock, the fact that I had angina… (P12, Female, Intervention)

Well, it never occurred to me that it would be angina, because I'd swam and that … (P1, Male, Control)

Within the intervention group, there was confusion around understanding some aspects of angina, such as whether a person could be said to still ‘have’ angina if they had undergone a revascularization procedure:

You haven't actually got angina once you've had the procedure, have you? I haven't got angina now. Well, I wouldn't say I've got angina… (P9 Female, Intervention)

### Dispelling myths

A positive element of the management programme for intervention participants was the opportunity to be better informed about angina rather than acting on inaccurate information, and the ability of the programme to give accurate information to partners and carers was also seen as a plus point:

I think it was good for her [wife] […] because, of course they worry and they try to cosset you a little bit and perhaps try to stop you from doing things that you want to do and which as it says you can do. […] If you just say no it's all right to do it, she's not going to believe you, but if she reads it you know it does help in that way. It's good that it included your next of kin or people that you know – and all the myths and things like that. (P8, Male, Intervention)

### Information about angina

Both the control and intervention groups reported receiving generally good written information about angina and its management. Control group members talked about a comprehensive booklet received from hospital among those who had undergone revascularization which was easy to follow and which they continued to consult. Leaflets from the British Heart Foundation (BHF) were rated as useful though somewhat repetitive. None of the control group mentioned any spoken advice that they had received from the angina specialist nurse (who had given them the written information).

Well the little booklets were a bit repetitive, but the big book [….] took you right through everything […] I'm still going back to it looking up – the exercises and all that. (P1 Male Control)

The intervention groups also found the BHF leaflets useful but commented mainly on their experience of the Angina Plan which was rated highly. Particularly useful features were the clarity and layout of information, making it easy to use. It was judged particularly valuable because it provided a comprehensive ‘checklist’ of ‘do's and don'ts’ and a diary system for logging of activity and progress. The diary system was seen as an aid to discipline but also as a source of encouragement. However, some participants suggested that whilst the diary system was useful, it may only encourage adherence to the programme for the limited duration of supported monitoring:

… having to fill the log in made me do a few things differently. […] I think having that discipline of making you do that, for me, certainly made me do things differently for a few months. (P6, Male, Intervention)

Participants in both the control and intervention groups generally reported not having supplemented their written information by seeking out other sources, preferring to rely on the information given by healthcare practitioners. However, one member of the intervention group had looked on the internet for information about heart problems.

### Medications

There was some confusion about the role of different angina medications in discussions in both the control and intervention groups. Some participants would have liked more information about medications to avoid whilst taking prescribed medication for angina and suggested that this might be a useful addition to the Angina Plan in future editions:

I can't see no improvement with them tablets I don't feel any different if I don't have them tablets or I do, but my doctor said like when you get 45–50-ish, you'd be better having them, so I don't know. (P4, Male, Control)

[…] that might be useful for the book. If you said, well you're going to be on certain medication, what tablets should you avoid? (P5, Male, Intervention)

### Lifestyle changes

Both control and intervention groups participants talked about the lifestyle changes that they needed to make, those they had already made, and efforts they were engaged in to effect changes, as well as the challenges that they faced in terms of health behaviour change:

You see it's all right them saying about your diet and all that, but with me, I work away from home and most of the time it's take away food and I can't help that. I try eating proper when I'm at home, but while you're away you've no chance. (P4, Male, Control)

Participants in the intervention groups were more mixed in their views about the challenges of making dietary changes. There was also a perception among intervention group participants that the dietary changes they had made according to advice in the programme had also been effective in changing wider family eating patterns:

In some cases it's rubbed off on the family - they've ate better because you have to eat more healthier. (P8, Male, Intervention)

In the intervention group, people appeared more generally engaged with exercise. For one participant, the programme had acted as a spur to resume exercise after a period of relative inactivity due to family commitments. However, there was some lack of clarity about the best types of exercise for angina. Participants perceived that they had received rather contradictory messages about exercise from healthcare practitioners, for example in relation to swimming:

I used to go swimming and they said, we don't want you to go into the pool because the change in temperature could bring on an attack and I said well, 2 years ago I was doing this on a daily basis and I never had an attack. So obviously they were being cautious. I felt that it was a bit negative in a way. (P7, Male, Intervention)

I remember the cardiologist after the procedure said to me about exercise and I'm sure that he said swimming was fine. (P5, Male, Intervention)

Participants felt that clearer guidelines on forms of exercise to pursue and avoid were needed to inform not only themselves but others too. For example, one participant in the intervention group had been a regular gym member but felt she had to withdraw from her usual keep fit class because the teacher was reluctant to permit a person with an angina diagnosis to participate. There was, however, a marked perception among intervention group members of the importance of maintaining fitness levels by exercising regularly.

The notion of ‘pacing’ tasks and activities arose in both control and intervention groups. Participants talked about the efforts they had made to incorporate pacing in their lives such as breaking up tasks into shorter activities with rests in between. The issue of relaxation arose in the intervention groups, based around perceptions of the relaxation CD that formed part of the Angina Plan. There were mixed views about the usefulness of the CD, roughly half of the intervention group finding it useful. Those who had found the CD helpful however had been able to use it for wider purposes such as to help with the management of pain associated with other conditions or in stressful situations:

I was in a restaurant and just towards the end of the meal I noticed my bag had gone and it had everything in, credit cards, camera, you know […] I said, ‘Right, there's nothing I can do about it. We'll go to the police station.’ They were saying, ‘Why are you so calm?’ ‘Because I've used my relaxation!’ (P12, Female, Intervention)

Among the half who did not find the relaxation exercise helpful, this was mainly attributed to the voice on the CD being perceived as irritating, or tending to induce sleepiness rather than relaxation.

Participants in the intervention group emphasized the importance of following the advice they had been given through the programme. There was a feeling among intervention focus groups that the Plan was so beneficial that it should be rolled out to others with angina, together with screening for heart problems and that it would also be valuable to anyone interested in looking after their health:

When I was last speaking to one of the nurses, she was saying to me that really this isn't a plan for angina, it's a plan for better living…and I think they might take the word angina out and possibly call it the Life Plan, or whatever. […] because then I think you can hand it to your partner and say you can do this as well, just because you think that you haven't got angina, doesn't mean to say you shouldn't follow what it says in here. So I think it's more of a Plan for Better Living. (P7, Male, Intervention)

### Impressions of care during the study

For the control group, the focus tended to be on medications and revascularization procedures as the most useful parts of their experiences and of most benefit to their health. The intervention group however made more extensive comments on their experiences during the study. There was general agreement that the Angina Plan had been invaluable and participants were glad to have been given the opportunity to take part. The opportunity to improve general fitness was a plus point for some, whilst others saw value in the encouragement the programme offered for them to prioritize looking after themselves. This appeared to be particularly marked for women:

I've coped much better than I actually thought I would have done [with a stressful life event]. […] I've had to look after myself, which I think women are not very good at doing. We put everybody else before ourselves and I feel guilty spending time on myself – that was one of the things that really did make me concentrate on that diary. (P13, Female Intervention)

### Participant perceptions of the lay-workers

Perceptions of the lay-workers facilitating the Angina Plan were extremely positive among intervention participants. Several elements were particularly appreciated such as providing encouragement, giving time, and being a trusted role model:

I had confidence in the fact that he'd been through it and that if I had any problems of any sort, I could raise them up with him. I didn't have to bother the doctor, you know, ring the doctor every five minutes, and there were some niggling things […] (P12, Female, Intervention)

The lay-worker was viewed as someone who would give precious time to participants, who was valued for their accessibility and viewed as highly approachable. The social aspect of having the lay-worker call to participants' homes was particularly valued and importantly enabled participants to feel cared for. A relationship had built up with the lay-worker and participants commented that they missed the contact at the end of the programme. Another positive element associated strongly with the lay-worker was that they were seen to provide an incentive for participants to act on the programme, adhere to it, and achieve their goals.

A common perception of the lay-worker was as someone who could be trusted to give the right information because they had been through a similar experience. The fact that lay-workers had also been living with angina and managing it successfully, or had a family member who had, reassured participants, normalized their experience and gave them confidence:

When I was going in for the angiogram… he talked me through the angiogram and that was very useful because you can read things on the internet but it's not the same as having somebody who's actually gone through it. (P13, Female, Intervention)

The only points of concern about the lay-worker role came from a single participant who expressed worries about the lay-worker's possible access to medical records and competence to advise on issues related to angina:

They [lay-workers] say well, I'm not a qualified doctor, I've just been trained in a support function. So how much access have you got to all my medical records and so on? That's a minor point, but even so, he's a man on the street basically, isn't he? … She [wife]… was worried that he was giving me prescriptions and advice about medical things when he wasn't a medical practitioner. And I said, no, he's just – he's been through it and he's sharing his experiences with me. (P7, Male, Intervention)

The same participant questioned whether the particular lay-worker assigned to him was an adequate ‘role model’ because the worker was perceived as overweight and by extension perhaps less qualified to give lifestyle advice.

## Discussion

Other studies have compared lay-health advisors with health professional advice in people with long-term conditions and found that they produced comparable results in people with asthma (Partridge *et al*. 2008) and in diabetes (Baksi *et al*. 2008). It should be noted that the study reported here did not compare like with like. The control group received one extra information session with the angina specialist nurse shortly after diagnosis, whereas the intervention group had ongoing support by home visit and telephone for 12 weeks, potentially enabling a deeper relationship to be formed.

Overall the Angina Plan, facilitated by lay-workers, was very well received by the participants involved in the study, which echoes previous research among people receiving the Angina Plan facilitated by nurses (Sykes *et al*. 2006). This study has demonstrated both similarities and differences between control and intervention groups. In line with previous literature (Furze & Lewin 2000, Tod *et al*. 2001), all participants reported low levels of prior knowledge about angina and feelings of shock upon diagnosis as many attributed their symptoms to other causes such as indigestion. The tendency to downplay or ignore bodily symptoms of illness has long been noted (Tod *et al*. 2001, Ryan & Zerwic 2003), and the process of ‘sanctioning’ bodily signs and symptoms of illness through discussion with others before seeking medical help (the ‘lay referral network’) is well known (Vassilev *et al*. 2011).

Participants' current understandings of living with angina were often marked by confusion about the persistence of the condition, characteristics of symptoms, and purpose of medication, which was apparent in both groups. This lack of clarity may suggest a ‘downside’ of the Angina Plan facilitator being a lay person. All participants received advice from a specialist nurse following diagnosis, and questions from intervention group participants could be referred to the nurses supervising the lay facilitators. However, on occasion, lay facilitators were unable to provide the depth of knowledge and information wanted by participants, particularly around medications, which the lay-worker training had emphasized as beyond their role. These findings suggest that a ‘mixed’ support approach involving both lay-workers and nurses may offer patients the opportunity to ask treatment or medication-specific questions.

**What is already known about this topic**

- People with stable angina are often excluded from cardiac rehabilitation pathways.
- Although nurse-facilitated self-management with the Angina Plan has been shown to reduce angina report while improving physical and psychological functioning, uptake in primary care has remained low.
- Lay-led self-management for long-term conditions has been suggested as a means of producing cost-effective improvements in health.

**What this paper adds**

- Participants in both the intervention and control arms of the study reported satisfaction with their care, although many were unclear about aspects of their condition and its treatment.
- Participants who received the Angina Plan intervention generally agreed that the contact provided by lay-workers was very useful in providing information, maintaining motivation, and facilitating change.
- On occasion, participants received contradictory advice from health professionals, which the lay-workers did not dispel or clarify, perhaps from fear of appearing to countermand health professional expertise.

**Implications for practice and/or policy**

- As patients continue to report a lack of knowledge about angina, there is still a need for services to provide detailed, consistent information about the diagnosis, treatment, and management of angina.
- Where nurses have limited time or resources to support self-management in people with angina, lay-workers facilitating the Angina Plan programme are likely to be positively received.
- As people with angina are likely to have needs for information and care that are beyond the scope of lay-workers, a clinical pathway that includes both forms of provision is more advisable than a lay-only format of self-management support.

Misconceptions about living with angina and their effect on health outcomes have been highlighted previously in the literature (Furze *et al*. 2003, 2005). A positive element of the management programme for intervention participants and their family/carers was the opportunity to be better informed about angina. Both groups demonstrated high levels of awareness about appropriate lifestyle changes and reported efforts to achieve them in terms of diet, exercise, and pacing of activity. This study did not assess whether the Angina Plan improved participants' perceptions of ability to change behaviour. Speechly *et al*. (2010) reported that people with heart disease may perceive behaviour change to be difficult, which may create a barrier to success in reducing risk. There was general satisfaction with cardiac services and information provided by healthcare practitioners, with particular value attached to staff attitudes such as interpersonal manner, professionalism, and caring. However, some participants experienced ambiguous or contradictory advice from health professionals, which the lay-workers may have felt unable to address due to the risk of countermanding health professional advice.

There was concern about the access of the lay-worker to medical records. This suggests that some participants may not have understood that the lay-worker was, as a member of health service staff, bound by confidentiality provisions. Better explanation for service users is required in order for them to be reassured about such novel forms of health service.

The programme evaluated here was a disease-specific, individual, home-based lay-led programme. The most famous lay-led programme is the Stanford Chronic Disease Self-Management Programme which is delivered in the UK as the Expert Patient Programme (EPP) (Department of Health 1999). A systematic review of the Expert Patient self-management interventions showed that they were successful in changing self-efficacy, but were unlikely to have significant clinical effects (Griffiths *et al*. 2007). A randomized trial of the EPP for people postmyocardial infarction (Barlow *et al*. 2009) found that there were few benefits to be gained above conventional cardiac rehabilitation. In a nested qualitative study in the same trial, however, the participants reported valuing the EPP, which they felt provided greater psychological support for coping with heart disease, and stronger motivation to achieve behaviour change (Barlow *et al*. 2009). These experiences and views of receiving lay support for self-management were similar to those reported by the intervention participants in this study.

### Limitations

This study has several limitations. Recruitment to the focus groups was relatively low by comparison to conventional standards; however, each group generated lively discussions and a rich data set. The sample of participants is a source of potential bias, because people who agreed to participate may have had only positive experiences of a high standard of care. Nonetheless, participants contributed discerning and analytical comments about their experiences of care and ideas for improvements to services. It may not be easy to get a representative sample for focus groups as people who are less articulate or have communication impairments may be discouraged from participating. However, this study was able to elicit successfully the views of one participant with impaired communication. It is also acknowledged that the participants in a focus group may not be expressing their own definitive individual view, because they are speaking in a specific context in which it may be difficult for the researcher to clearly identify an individual message. It is acknowledged further that while including only one focus group for control participants may not have allowed true data saturation to be reached for this group, the primary focus of the study was to explore experiences of participants in the lay-facilitated angina management group.

## Conclusion

There is still a need for services to provide detailed, consistent information about the diagnosis, treatment, and management of angina. This research suggests that the Angina Plan facilitated by lay-workers would be positively received by patients. However, as a fully lay-facilitated programme would not be able to address more complex needs for information and support, there is an imperative that nurses are also skilled in the cognitive-behavioural techniques involved in successful self-management support. Further research into cooperative working between nurses and lay-workers in the delivery of patient-centred care is much needed, particularly in their potential to deliver a fully holistic model of care within constrained health budgets.
